# Predictors of a follicular nodule (Thy3) outcome of thyroid fine needle aspiration cytology among Saudi patients

**DOI:** 10.1186/s13104-017-2943-8

**Published:** 2017-11-23

**Authors:** Imad R. Musa, Mukhtar El khatim Ahmad, Fayez Salim Al Raddady, Wesal Rabih Al Rabih, Elsayed M. Elsayed, Gussay Badawi Mohamed, Gasim I. Gasim

**Affiliations:** 1 Armed Forces Hospital, Dhahran, Kingdom of Saudi Arabia; 2 Faculty of Medicine, Al-Neelain University, Khartoum, Sudan

**Keywords:** Thyroid fine needle aspiration cytology, Saudi Arabia, Solitary nodule, Multinodular goitre

## Abstract

**Objective:**

A retrospective study was performed to evaluate predictors of thyroid fine needle aspiration cytology (FNAC) outcomes among Saudis with a thyroid nodule. Socio-demographic data, thyroid function status, thyroid parameters, ultrasound and cytology results were collected from 269 files of patients with thyroid nodules.

**Result:**

The patients’ age was 40 ± 1.4 years (mean ± SD), and the mean body mass index (BMI) was 30.3 ± 1.2 kg/m^2^. The thyroid statuses were euthyroid (85.5%), hypothyroidism (7.4%) and hyperthyroidism (7.1%). Young age, an absence of irradiation history, and multinodular goitre were protective against Thy3 [(OR = 0.05, CI = 003–0.6, P = 0.024), (OR = 0.4, CI = 0.2–0.8, P = 0.012) and (OR = 2.5, CI = 1.2–5.3, P = 0.016), respectively]; a lower FT3 was protective against Thy4 (OR = 0.4, CI = 0.2–0.99, P = 0.046), the absence of cervical lymphadenopathy was associated with Thy2 (OR = 2.7, CI = 1.4–5, P = 0.001), and a solid nodule was associated with Thy2 and Thy3 [(OR = 1.2, CI = 0.3–0.97, P = 0.040) and (OR = 2.2, CI = 1–4.8, P = 0.039), respectively]. In a multivariate analysis, younger age, multinodular goitre, an absence of irradiation history and cervical lymphadenopathy were protective against Thy3 [(OR = 0.04, 95% CI = 0.002–0.96, P = 0.047), (OR = 2.4, 95% CI = 1.0–5.60, P = 0.039), (OR = 0.4, 95% CI = 0.16–0.94, P = 0.036) and (O R = 0.39, 95% CI = 1–5.6, P = 0.039), respectively]. In summary, younger age, multinodular goitre, the absence of an irradiation history and cervical lymphadenopathy were protective against Thy3 in a thyroid nodule.

## Introduction

A thyroid nodule is an abnormal lesion within the thyroid tissue, according to The American thyroid association (ATA) [1]. The incidence of thyroid nodules has been reported to be approximately 0.1% per year [2, 3]. The prevalence of palpable thyroid nodules ranges from 1 to 7% in different clinical data [1]. The frequency of detecting thyroid nodules increases with age and in areas where low iodine intake is observed. Thyroid nodules harbour a risk of cancer of approximately 5–10%, and therefore it’s important to exclude malignancy on detecting them [4]. Thyroid cancer has been reported to be on the rise worldwide, and Saudi Arabia is not an exception [1, 5, 6]. Females are known to have a higher risk of thyroid cancer than males, particularly in the age group 30–49 [7]. It has been described that the extremes of age are associated with an increase in thyroid cancer [4]. Furthermore, exposure to radiation is regarded as a risk factor for thyroid malignancy, particularly if it occurred during childhood; therefore, proper history and evaluation is of paramount importance for a child with a thyroid nodule [1].

Assessment of serum thyroid function tests should be part of the routine package of tests performed for all patients exhibiting thyroid nodules. Thyroid stimulating hormone (TSH) is an independent risk factor that predicts malignancy in a thyroid nodule. Boelaert et al. [8] described a prevalence of malignancy of 29% among those who had a serum TSH level higher than 5.5 mU/L, while it was only 2.8% among those with a serum TSH of < 0.4 mU/L. The American thyroid association has made a recommendation to use some risk factors in the evaluation of the thyroid nodule, such as a first-degree relative, rapid nodule growth, vocal cord paralysis, cervical lymphadenopathy, and whether the nodule is attached to surrounding tissues [1]. Ultrasound is a robust tool in the detection of a thyroid nodule, with the ability to determine the character of the nodule (solid or cystic, solitary or dominant nodule in a multinodular goitre), as well as the size and cervical lymph node features.

Characteristic sonographic features on a nodule have been used as predictors of malignancy. Some features of thyroid nodules, such as being taller than wide on a transverse view, the presence of infiltrative margins, an irregular outline, increased vascularity, hypo-echogenicity and micro-calcification, are presumed to predict cancer, despite the fact that none of these features is pathognomonic, either alone or in combination, to diagnose a nodule as malignant. Nevertheless, papillary and follicular thyroid cancers may adopt different features on an ultrasound scan [9]. Being a solid mass without cystic changes has been demonstrated in papillary carcinomas and follicular carcinomas [9]. On the other hand, a thyroid uptake scan is not routinely recommended unless a toxic nodule is a likely possibility in the case of a very low TSH, a value which deems fine needle aspiration cytology (FNAC) unnecessary since the nodule is always benign [1].

Historically, fine-needle aspiration (FNA) for cytological evaluation of thyroid cancer was originally performed at the New York Memorial Hospital for Cancer and Allied Diseases in 1930 by Martin and Ellis [10]. Since the 1950s, it has been adopted as a safe and cost-effective method of diagnosis [11]. Recently, some studies regarded fine-needle aspiration biopsy as the most reliable diagnostic tool for detecting malignancy in thyroid nodules, with a sensitivity and specificity that exceeds 90% [1, 12]. Studies have shown that the size of the thyroid nodule may not influence the outcome of the thyroid FNAC [13, 14]. This method of clinical investigation is now practised globally and is considered to be the cornerstone for a thyroid nodule evaluation [15, 16]. Despite the wide use and importance of such a robust diagnostic tool worldwide, there are few published data from the whole region. Taking into consideration the impact of genetics and their interaction with environmental factors, the current study aims to determine predictors that may influence the outcomes of thyroid fine needle aspiration cytology among Saudi subjects in the Armed Forces Hospital of King Abdu Aziz.

## Main text

### Methods

A retrospective study was conducted at the Armed Forces Hospital of King Abdul Aziz Airbase in Dhahran during the period of 1st January 2010 through 31st December 2016. The sample size was based on the size of similar studies elsewhere [17, 18]. The records of two hundred and sixty-nine adult Saudi patients with documented thyroid nodules who underwent fine needle aspiration cytology were retrieved. Records of non-Saudis, those with incomplete data, patients who underwent partial thyroid surgery without prior thyroid FNAC, patients diagnosed with thyroid cancer from lymph node biopsy and patients with known thyroid malignancy were excluded. The socio-demographic data was gathered using a data collection sheet. Ultrasound reports were used to determine the thyroid gland size, the nodule size (≤ 1 cm), the number of nodules (solitary or multinodular), the character of the nodule (solid, cystic or mixed) and the presence of enlarged cervical lymph nodes. After the application of a local anaesthetic under aseptic conditions, a 22-gauge needle with a 10-mL syringe was used. The tip of the needle was targeted to areas presumed to contain the most cellular material of the thyroid nodule while continuous low pressure suction was applied simultaneously with a to-and-fro movement of the needle within the lesion to get the material in the needle hub. The histology slides were prepared as soon as the specimens were drawn and labelled. A final cytopathology report was obtained after studying each cell block. The thyroid fine needle aspiration cytology outcome was reported using the modified Thy classification as shown in Table 1. Exposure to any sort of radiation was documented. The thyroid status (euthyroid, hypothyroidism and hyperthyroidism), thyroid parameters [TSH, Free T4(FT4), and Free T3(FT3)] were recorded just before the procedure of thyroid FNAC.

**Table 1:** Shows the modified Thy Classification

Thy1: non-diagnostic or inadequate sample	
Thy2: non-neoplastic or benign (features consistent with a nodular goiter or thyroiditis)	
Thy3: all follicular lesions. (classified as a follicular lesion/suspected follicular neoplasm)	
Thy4: abnormal, suspicious of malignancy (suspicious, but not diagnostic, of papillary, medullary or anaplastic carcinoma or of lymphoma)	
Thy5: diagnostic of malignancy (unequivocal features of papillary, medullary or anaplastic carcinoma, or of lymphoma or of metastatic tumor)	

### Statistics

Data were entered into SPSS for Windows V.24.0, (SPSS Inc., Chicago, IL, USA) which was used for analysis. After checking data for normality of distribution, an ANOVA was used for normally and non-normally distributed data after log transforming the non-normally distributed variables. Chi square was used to analyse the categorical data. Continuous data were presented in the form of the mean ± SD for normally distributed data or the median in the case of non-normally distributed data. The geometric mean was calculated in the case of non-normally distributed data that had undergone log transformation, while the categorical data was presented in the form of proportions. Factors predicting the outcome of the FNAC were entered into a multinomial logistic regression model if their p value was 0.05 or less. This multivariate logistic model included the following variables: age, BMI, the number of thyroid nodules, the thyroid nodule character, the thyroid hormonal profile apart from FT3, exposure to radiation, and cervical lymph nodes. A P value less than 0.05 was considered statistically significant.

### Results

Two hundred and sixty-nine subjects were enrolled in the study, out of which 34 (12.6%) were males and 235 (87.4%) were females. Their mean ± SD age was 40 ± 1.4 years, while their mean ± SD body mass index (BMI) was 30.3 ± 1.2 kg/m^2^. The distribution of cases according to the Thy classification as shown by the fine needle aspiration cytology was Thy-1 111 (41.3%), Thy-2 107 (39.8%), Thy-3 38 (14.1%), and Thy-4 13 (4.8%), and for Thy-5, no case was reported (0.0%).

Euthyroid cases formed the majority (85.5%), with hypothyroidism forming 7.4% and hyperthyroidism 7.1%.

A significant association was found between thyroid FNAC and age (P = 0.017), BMI (P = 0.002), thyroid function parameters (TSH and FT4) with a P value = 0.002 for each, FT3 (P = 0.005), radiation exposure (P = 0.025), number of nodules (P = 0.006), character of the nodule (P = 0.001) and absence of associated enlarged cervical lymph nodes (P = 0.010).In contrast, gender (P = 0.090), the size of the thyroid gland (P = 0.912), thyroid status (P = 0.584) and the thyroid nodule size (P = 0.318) had no association with FNAC (Table 2).

**Table 2:** Main patient’s characteristics

Variables	Thy1 N (111)	Thy2 N (107)	Thy3 N (38)	Thy4 N (13)	P value
Gender					
Male no. (%)	17 (15.3)	15 (14)	0 (0)	2 (15.4)	0.090
Female no. (%)	94 (84.7)	92 (86)	38 (100)	11 (84.6)	
Age in years mean (sd)	39.8 (1.4)	39.8 (1.4)	34.7 (1.3)	41.7 (1.4)	0.017
BMI kg/m^2^ mean (sd)	31.6 (1.2)	31.6 (1.2)	31.6 (1.2)	31.6 (1.2)	0.002
Thyroid status					
Euthyroid N (%)	94 (84.7)	89 (83.2)	36 (94.7)	11 (84.6)	0.584
Hypothyroidism N (%)	7 (6.3)	11 (10.3)	1 (2.6)	1 (7.7)	
Hyperthyroidism N (%)	10 (9)	7 (6.5)	1 (2.6)	1 (7.7)	
Thyroid gland size					
Normal N (%)	12 (10.8)	9 (8.4)	3 (7.9)	1 (7.7)	0.912
Enlarged N (%)	99 (89.2)	98 (91.6)	35 (92.1)	12 (92.3)	
The number of thyroid nodules					
Solitary N (%)	34 (30.6)	27 (25.2)	20 (52.6)	7 (53.8)	0.006
Multi-nodular N (%)	77 (69.4)	80 (74.8)	18 (47.4)	6 (46.2)	
The thyroid nodule character					
Solid N (%)	34 (30.6)	19 (17.8)	20 (52.6)	7 (53)	0.001
Cystic N (%)	12 (10.8)	16 (15)	1 (26.3)	1 (7.7)	
Mixed	65 (58.6)	72 (67.3)	17 (44.7)	5 (38.5)	
The thyroid nodule size					
Less than 1 cm N (%)	5 (4.5)	2 (1.9)	0 (0)	1 (7.7)	0.318
More than 1 cm N (%)	106 (95.5)	105 (98.1)	38 (100)	12 (92.3)	
The thyroid hormonal profile					
TSH mean (sd)	1.2 (4.2)	1.2 (3.3)	7.9 (2.9)	1.5 (2.9)	0.002
T3 mean (sd)	4.3 (1.2)	4.6 (1.3)	4.5 (1.2)	3.9 (1.4)	0.005
T4 mean (sd)	12.6 (1.2)	13.2 (1.2)	12.9 (1.2)	12.6 (1.1)	0.002
Exposure to radiation					
Yes N (%)	90 (81.1)	86 (80.4)	23 (60.5%)	12 (78.4)	0.025
No N (%)	21 (18.9)	21 (19.6)	15 (39.5)	1 (21.6)	
Cervical lymph nodes					
Normal N (%)	65 (58.6)	85 (79.4)	24 (63.2)	9 (69.2)	0.010
Enlarged N (%)	46 (41.4)	22 (20.6)	14 (36.8)	4 (30.8)	

Univariate analysis showed young age, an absence of a history of exposure to radiation and multinodular goitre to be protective against Thy3 [(OR = 0.05, CI = 003–0.6, P = 0.024), (OR = 0.4, CI = 0.2–0.8, P = 0.012) and (OR = 2.5, CI = 1.2–5.3, P = 0.016), respectively], a lower FT3 was protective against Thy4 (OR = 0.4, CI = 0.2–0.99, P = 0.046), the absence of cervical lymphadenopathy is associated with Thy2 (OR = 2.7, CI = 1.4–5, P = 0.001) and a solid nodule was found to be a risk factor for the development of Thy2 and 3 [(OR = 1.2, CI = 0.3–0.97, P = 0.040) and (OR = 2.2, CI = 1–4.8, P = 0.039), respectively] (Table 3).

**Table 3:** Showing the univariate and multivariate logistic regression analyses of FNAC outcome where FNAC Thy1 was the reference

Variables	Thy2 (N = 107)				Thy3 (N = 38)				Thy4 (N = 13)			
		OR	CI	P		OR	CI	P		OR	CI	P
Univariate												
Age in years mean (SD)	39.8 (1.4)	1.6	0.2–10.6	0.652	34.7 (1.3)	0.05	0.003–0.6	0.021	41.7 (1.4)	2.3	0.04–150.3	0.691
No TN												
SN No (%)	27 (25.2)	0.8	0.4–1.4	0.375	20 (52.6)	2.5	1.2–5.3	0.016	7 (53.8)	2.6	0.8–8.5	0.101
MNG N (%)	80 (74.8)				18 (47.4)				6 (46.2)			
ER												
Yes N (%)	86 (80.4)	0.9	0.5–1.9	0.895	23 (60.5)	0.4	0.2–0.8	0.012	12 (78.4)	2.8	0.5–22.7	0.335
NO N (%)	21 (19.6)				15 (39.5)				1 (21.6)			
CLN												
Normal N (%)	85 (79.4)	2.7	1.4–5	0.001	24 (63.2)	1.2	0.6–2.6	0.618	9 (69.2)	1.6	0.5–5.5	0.461
Enlarged N (%)	22 (20.6)				14 (36.8)				4 (30.8)			
Gender												
Male N (%)	15 (14)	0.9	0.4–1.9	0.787	0 (0)	NA			2 (15.4)	1	0.2–4.9	0.995
Female N (%)	92 (86)				38 (100)				11 (84.6)			
Thyroid gland size												
Normal N (%)	9 (8.4)	0.8	0.3–1.9	0.549	3 (7.9)	0.7	0.2–2.7	0.608	1 (7.7)	0.78	0.1–5.9	0.730
Enlarged N (%)	98 (91.6)				35 (92.1)				12 (92.3)			
Nodule character												
Solid N (%)	19 (17.8)	1.2	0.3–0.97	0.040	20 (52.6)	2.2	1–4.8	0.039	7 (53)	2.7	0.8–9	0.114
Cystic	16 (15)				1 (26.3)				1 (7.7)			
		1.2	0.5–2.7	0.658		0.3	0.04–2.6	0.288		1.1	0.1–10	0.944
Mixed	72 (67.3)				17 (44.7)				5 (38.5)			
Nodule size												
> 1 cm	2 (1.9)	0.4	0.1–2.1	0.285	0 (0)	NA			1 (7.7)	1.8	0.2–16.4	0.617
< 1 cm	105 (98.1)				38 (100)				12 (92.3)			
Thyroid hormones												
TSH mean (sd)	1.2 (3.3)	1	0.9–1.2	0.414	7.9 (2.9)	0.9	0.7–1.2	0.583	1.5 (2.9)	1	0.9–1.2	0.688
T3 mean (sd)	4.6 (1.3)	1.2	0.8–1.8	0.347	4.5 (1.2)	1.1	0.7–1.7	0.626	3.9 (1.4)	0.4	0.2–0.99	0.046
T4 mean (sd)	13.2 (1.2)	1.1	0.97–1.2	0.159	12.9 (1.2)	1	0.9–1.2	0.593	12.6 (1.1)	1	0.8–1.3	0.954
BMI mean (sd)	31.6 (1.2)	1.5	0.1–40.6	0.809	31.6 (1.2)	1.7	0.02–163.5	0.826	31.6 (1.2)	0.05	4.248E−50.1	0.401
Multivariate												
Age in years mean (SD)	39.8 (1.4)	1.6	0.2–13.2	0.674	34.7 (1.3)	0.04	0.002–0.96	0.047				
No TN												
SN No (%)	27 (25.2)	0.7	0.3–1.3	0.239	20 (52.6)	2.4	1.0–5.6	0.039				
MNG N (%)	80 (74.8)				18 (47.4)							
ER												
Yes N (%)	86 (80.4)	1.0	0.5–2.1	0.999	23 (60.5)	0.4	0.16–0.94	0.036				
NO N (%)	21 (19.6)				15 (39.5)							
CLN												
Normal N (%)	85 (79.4)	3.2	1.6–6.1	< 0.001	24 (63.2)	0.39	1–5.6	0.039				
Enlarged N (%)	22 (20.6)				14 (36.8)							

A multivariate analysis showed that younger age, multinodular goitre, absence of a history of radiation exposure and an absence of lymphadenopathy provide a favourable prognostic effect against acquiring Thy3 in thyroid nodules [(OR = 0.04, 95% CI = 0.002–0.96, P = 0.047), (OR = 2.4, 95% CI = 1.0–5.60, P = 0.039), (OR = 0.4, 95% CI = 0.16–0.94, P = 0.036) and (OR = 0.39, 95% CI = 1–5.6, P = 0.039), respectively] (Table 3).

### Discussion

Thy3 cases formed 14.1% of the cases in the current study; a similar finding has been described in the United Kingdom [19]. Al-Shraim et al. [20] described a greater Thy3 percentage (29.7%) of their cases in the study conducted in the Southwestern region of Saudi Arabia; however, the area is mountainous and known to be endemic for iodine deficiency [21]. It is worth mentioning that most of the thyroid cancer studies focused on the incidence of the disease, and none of these studies addressed the predictors of the outcome. One of the main findings in this study is the significant association between fine needle aspiration cytology and age, where the association showed that younger age has a protective effect against the development of Thy3, although this association is weak. Similar studies have shown wide variations and conflicting results. While some of these studies showed an association with the extremes of age (less than 20 years or more than 70 years) [4], others considered age as a low malignancy-risk factor [22, 23]; however, no association was found [24]. Such variation can be explained by the differences in the genetic backgrounds of the study groups as well as differences in the influence of environmental factors. In this regard, our patients are living in an oil industry area where radiation hazards cannot be totally excluded. Furthermore, the gulf area is still a hot battlefield where depleted uranium is frequently used. Such observations might be taken into account as explanations for the recently reported increase in thyroid cancer, where it formed 8.1% of new cancer cases in 2012, according to The Saudi Cancer Registry [25].

Moreover, a solitary nodule was found to be more likely associated with Thy3 than a multinodular goitre. Such a result is in accordance with published data where a higher prevalence of thyroid malignancy has been linked to a solitary nodule rather than to a multinodular goitre [26–28]. In contrast to this finding, other authors showed that the number of thyroid nodules cannot predict the histological outcome of a thyroid fine needle aspiration cytology [29, 30]. Others claim that the overall risk of cancer within a gland with a solitary nodule is similar to that of a multinodular gland [31].

Accordingly, dealing with each thyroid nodule separately and irrespectively of its number is a wise attitude for proper evaluation. The current study showed that the absence of a radiation history has a protective effect against the development of Thy3. Exposure to radiation during childhood is considered to be a predictor for thyroid malignancy, including follicular neoplasms, because the sensitivity of the thyroid gland to radiation is significantly greater [32]. A recent study from Belarus described such an association [33]. However, the risk of getting thyroid cancer may persist for many decades after radiation exposure, and an annual physical examination should be thus encouraged as a thyroid cancer screening strategy [34]. Radiation exposure from CT scans is estimated to be small but can lead to a significant increase to the overall risk of thyroid nodule related cancer [35, 36]. However, others reported different findings [37]. Such conflicting results might be explained by the variation in the genetic background between the different groups or the difference in radiation dose and may be due to the nutritional background, as deficiency in iodine can play such a perpetuating role. Enlarged cervical lymph nodes were found to predict the presence of follicular neoplasm (Thy3 subgroup). A positive predictive value of 68% with an 82% sensitivity and 90% specificity for thyroid cancer has been demonstrated in cases with associated enlarged cervical lymph nodes [38]. Enlarged cervical lymph nodes can represent an indicator for a malignant aetiology if found on the same side as a thyroid nodule and in the presence of some suggestive features, such as micro calcifications, increased vascularity, cystic changes, and rounded shape [39].

Other factors included in this study either showed no association using a univariate analysis or were found to be confounders when entered into the multivariate analysis.

### Conclusion

The current study showed that younger age, multinodular goitre, the absence of a history of exposure to radiation, and an absence of lymphadenopathy provide protective evidence against acquiring Thy3 in thyroid nodules, but a solitary nodule does not provide evidence of protection.

## Limitations

The limitations of this study were that both thyroid ultrasound and FNAC are subject to variation, based on the experience and skills of the performing person. As a retrospective study, it suffered from insufficiency of records in the following areas: thyroid scintigraphy and detailed features of thyroid nodules, family history of thyroid cancer, and the status of autoimmune thyroid antibodies as risk factors.

## Abbreviations

FNAC: fine needle aspiration cytology
BMI: body mass index
TSH: thyroid stimulating hormone
FT4: free T4
FT3: free T3
TN: thyroid nodule
SN: solitary nodule
MNG: multinodular goitre
ER: exposure to radiation
CLN: cervical lymph nodes
NA: not available
